# Revisiting Non-BRCA1/2 Familial Whole Exome Sequencing Datasets Implicates *NCK1* as a Cancer Gene

**DOI:** 10.3389/fgene.2019.00527

**Published:** 2019-06-04

**Authors:** Jie Yin, Kai Wu, Qingyang Ma, Hang Dong, Yufei Zhu, Landian Hu, Xiangyin Kong

**Affiliations:** State Key Laboratory of Medical Genomics, Institute of Health Sciences, Shanghai Jiao Tong University School of Medicine and Shanghai Institutes for Biological Sciences, Chinese Academy of Sciences, Shanghai, China

**Keywords:** breast cancer, non-BRCA1/2, *NCK1*, predisposition gene, invasion

## Abstract

Through linkage and candidate gene screening, many breast cancer (BC) predisposition genes have been identified in the past 20 years. However, the majority of genetic risks that contribute to familial BC remains undetermined. In this study, we revisited whole exome sequencing datasets from non-BRCA1/2 familial BC patients, to search for novel BC predisposition genes. Based on the infinite mutation model, we supposed that rare non-silent variants that cooccurred between familial and TCGA-germline datasets, might play a predisposition contributing role. In our analysis, we not only identified novel potential pathogenic variants from known cancer predisposition genes, such as *MRE11*, *CTR9* but also identified novel candidate predisposition genes, such as *NCK1*. According to the TCGA mRNA expression dataset of BC, *NCK1* was significantly upregulated in basal-like subtypes and downregulated in luminal subtypes. *In vitro*, NCK1 mutants (D73H and R42Q) transfected MCF7 cell lines, which attributed to the luminal subtype, were much more viable and invasive than the wild type. On the other side, our results also showed that overall survival and disease-free survival of patients with *NCK1* variations might be dependent on the genomic context. In conclusion, genetic heterogeneity exists among non-BRCA1/2 BC pedigrees and *NCK1* could be a novel BC predisposition gene.

## Introduction

Breast cancer (BC) is the most malignant cancer type, affecting women worldwide (30%) and is the secondary cause of death in women (14%) (Siegel et al., 2018). Although most BC patients are sporadic, about 10–15% of BC s show familial aggregation (Kiiski et al., 2014; Lynch et al., 2015). High penetrance genes, such as *BRCA1* and *BRCA2*, contribute about 20% to the etiology of familial BC (Mavaddat et al., 2010; Rizzolo et al., 2011; Melchor and Benitez, 2013). While linkage analyses failed to identify any compelling evident region of linkage in non-BRCA1/2 BC pedigrees (Antoniou and Easton, 2006). According to candidate gene screening, other high or moderate penetrance genes, such as *TP53*, *PALB2*, *STK11*, *ATM*, and *CHEK2* have been identified (Stratton and Rahman, 2008; Melchor and Benitez, 2013). With the application of Whole Exome Sequencing (WES), several novel BC predisposition genes have been identified from BC pedigrees, which further confirms that non-BRCA1/2 familial BC is highly heterogeneous.

An evaluation of potential predisposition roles of germline variants is challenging. First, to distinguish disease-causative variants from the non-pathogenic ones during WES analysis usually involves a series of filtering steps, including *in silico* prediction; however, such filtering steps might cause over-filtering or be misleading (Bamshad et al., 2011). For instance, on one hand, *in silico* predictions might not be sensitive enough to detect all deleterious or damaging variants; on the other hand, the *in silico* predicted damaging variants might not be clinically pathogenic (Rahman, 2014). Second, to identify predisposition factors usually starts with an inspection of familial aggregation datasets, followed by a case-cohort confirmation (Kiiski et al., 2014); however, variants may be misclassified as having a uncertain significance due to their extreme rarity and heterogeneity. The efficiency of predisposition gene identification cannot be promoted significantly by simply increasing sample size. Third, incidental findings, which are not related to the observed phenotype of the patient, also complicate the analysis of the WES result (Kohane et al., 2006).

The American College of Medical Genetics and Genomics-Association for Molecular Pathology (ACMG-AMP) based guidelines have been widely used in variant classification (Hampel et al., 2015). Recently, ACMG-AMP-based variant classification rules have also been used in familial BC (Maxwell et al., 2016) and pan-cancer datasets (Huang et al., 2018). Of note, a co-segregation status of a germline variant is also important for variant classification (Jarvik and Browning, 2016). Pan-cancer studies have provided valuable sources to inspect tumor initiation and progression (Weinstein et al., 2013). An integrative analysis of germline and somatic variants could help to decipher tumor progression (Kanchi et al., 2014). We supposed that the co-occurrence between non-silent familial co-segregation variants and TCGA derived germline datasets could provide supporting evidence for a predisposition. Furthermore, pan-cancer datasets would also provide additional clues and evidence. Given that, we reanalyzed the WES datasets including 10 familial non-BRCA1/BRCA2 BC pedigrees (Gracia-Aznarez et al., 2013; Hilbers et al., 2013), manually evaluated variants as recommended (Hampel et al., 2015), and performed data mining on pan-cancer datasets.

In our analysis, some recently published BC predisposition genes, including *MRE11* (Bartkova et al., 2008), *CTR9* (Hanks et al., 2014), were recalibrated in our results, but were missed in the original publication. In addition, we identified novel cancer predisposition genes, such as *NCK1*. *NCK1* encodes the cytoplasmic adaptor protein NCK1, which contains Src homolog2 and 3 (SH2 and SH3) domains. As an adaptor, NCK1 mediates multiple signals from receptors, including EGFR, PDGFR, to downstream effectors and the overexpression of Nck in the NIH 3T3 cell line showed oncogenic features (Li et al., 1992). In mammalians, most Nck1 effectors are involved in cytoskeletal dynamics (Li et al., 2001). For instance, Nck1 is involved in actin cytoskeletal remodeling via the WASp/Arp2/3 complex, which in turn causes the polarization and directional migration of the cell (Lapetina et al., 2009). Interestingly, the mutation *NCK1* (p.D73H) identified from the BC pedigree (F2887) is located in an N-WASP activation motif (Okrut et al., 2015). Therefore, we supposed that *NCK1* (p.D73H) might impact cell invasion. MCF7 cell lines, which are non-invasive, transfected with *NCK1* mutants and were much more viable and invasive, *in vitro*. In conclusion, our results support that *NCK1* could be a candidate cancer predisposition gene.

## Materials and Methods

### Whole Exome Sequencing Datasets

In this study, we reanalyzed WES data of non-*BRCA1/BRCA2* BC pedigrees (Gracia-Aznarez et al., 2013; Hilbers et al., 2013). Ten pedigrees with at least two independent patients applied to whole exome sequencing were involved in this study. The raw data of pedigrees (2887, 3311, RUL36, and RUL153) are available at National Centre for Biotechnology Information (NCBI) Sequence Read Archive (SRA) database (Project ID: PRJEB3235). The raw data of pedigrees (NIJM6, NIJM8, RUL39, RUL70, RUL79, and RUL154) were transmitted with permission. The authority of the datasets about those pedigrees belongs to the original authors.

### Variant Calling, Annotation, and Evaluation

We mapped the WES reads against the human reference genome (hg19) using BWA *mem* mode, with parameters set as default (Li and Durbin, 2009) and preprocessed as recommended (McKenna et al., 2010). Mindful that highly quality off-target variants could be identified from WES (Guo et al., 2012), we generated all exon regions with flanking 100 bp via UCSC Table browser supplied to GATK for variant calling. We combined VQSR (Variant Quality Score Recalibration) and a hard filters to filter out potential false positive variants. The parameters are summarized in the Supplementary Table S1. The variants were then annotated with ANNOVAR (Wang et al., 2010) and classified as recommended (Hampel et al., 2015). The databases involved in annotation and the variant classification methods are summarized in the Supplementary Table S1.

### Vector Construction, Cell Culture, and Transfection

Full-length *NCK1* was cloned from pLX304 to MSCV-5′HA (3×). We generated point mutants of *NCK1* (p.D73H and p.R42Q) via site-directed mutagenesis with primers designed by Primer X^1^. All the vectors were confirmed via Sanger sequencing. For lentivirus production, the *NCK1* mutants containing MSCV vectors were co-transfected with pCMV-VSVg and GAG/pol plasmids into 293FT cells by Lipo2000. Cell lines were cultured at 37°C under 5% CO_2_ in DMEM, high glucose medium (Gibco) with 10% (v/v) fetal bovine serum (FBS; Gibco) and penicillin G (100 U/ml, Gibco) and streptomycin (100 ug/ml, Gibco).

### Cell Viability Assay and Transwell Invasion Assay

Cell viability was assessed with MTT colorimetric assay (Ameresco), at time periods of 6 days. The optical absorbance was measured at 562 nm on a spectrophotometer (Biotek), and the reference wavelength at 630 nm. All the experiments were performed in triplicate and repeated three times. Cell invasion assays were performed using 24-well transwell (8 μm pore, Corning) that were coated with 1:10 diluted Matrigel Matrix (BD Biosciences). A total of 2 × 10^4^ cells, in 200 μL of serum-free DMEM medium, were added into the upper transwell chamber, and 500 μL of 10% FBS DMEM medium containing 1 μg/mL EGF was added into the lower chamber. After incubation for 48 h, the cells were fixed in 4% paraformaldehyde and stained with 0.1% crystal violet. The cell images were taken at five random microscopic fields (Olympus, 10×). All experiments were repeated three times. The Student’s *t*-test was used to test whether the difference was significant.

### *NCK1* Mutation Analysis

TCGA-germline variants were retrieved by subtracting the non-TCGA variants (ExAC-non-TCGA) from the whole dataset (ExAC) (Lek et al., 2016). Pan-cancer somatic mutations of *NCK1* were retrieved from cBioportal (Cerami et al., 2012). We performed a hotspot analysis on *NCK1* somatic mutations via the R package DominoEffect (Buljan et al., 2018). The flanking regions were determined after normalizing the gene length and impaired residues by function *calculate boundary* (Buljan et al., 2018). In order to evaluate substitution tolerance of *NCK1* mutations, position specific score matrix (PSSM) was generated by PSI-BLAST (Altschul et al., 1997). For a given missense mutation, we obtained the score difference between the mutation and wild type residue: ΔS = S _mutation_ – S _wild–type_. We generated 10,000 sets of three random mutations of *NCK1* and evaluated the mean score for each set.

### *NCK1* Mutation Burden Analysis

To perform mutation burden analysis of *NCK1* germline mutations in a cancer-cohort and normal controls, we retrieved the allele count and allele number of corresponding *NCK1* mutations from the general cohort, control-cohort, non-cancer cohort collected from the Genome Aggregation Database (genomAD) (Karczewski et al., 2019). The cancer-cohort specific allele count and allele number of *NCK1* mutations was obtained by deducting the non-cancer cohort from the general cohort. A Fisher test was used to test the occurrence of non-silent mutations in *NCK1* across the cohorts mentioned above.

### *NCK1* Expression Analysis

As described before (Chen et al., 2016), the mRNA expression level in *NCK1* (RNA-seq V2) of 99 tumor-normal matched BC samples were retrieved from the Cancer Genome Atlas database (Weinstein et al., 2013) and the RSEM normalized result were applied to the downstream analysis. Among them, 95 patients owned inferred PAM50 subtypes (Netanely et al., 2016).

## Results

### Re-evaluation Variants Identified From Familial Breast Cancer Patients

We reanalyzed published Whole Exome Sequencing datasets from 10 non-*BRCA1/2* BC pedigrees (Gracia-Aznarez et al., 2013; Hilbers et al., 2013). Two samples per pedigree were applied to whole exome sequencing, and the kinship of the samples varied from 0.016 to 0.25 (Table 1). We set those rare non-silent variants, shared between patients per pedigree, as candidate co-segregated ones. To reduce incidental findings, we first focused on the genes that had been assigned with pathogenic supporting evidence (Supplementary Table S1), especially the known cancer predisposition genes (Rahman, 2014). Second, we filtered for variants with uncertain clinical significance, which must show in both the familial and TCGA germline dataset. The detailed variant filtering and classification parameters are summarized in Supplementary Table S1.

**Table 1:** Candidate predisposition genes identified from non-BRCA1/2 pedigrees.

Pedigree	Kinship	Evaluation	Genotype	Gene	Type	Transcript	Exon	HGVS(.c)	HGVS(.p)
F3311	0.0625	Likely pathogenic	Het	*MRE11*	Missense	NM_005591.3	Exon 3	c.94A > G	p.R32G
NIJM6	0.25	Likely pathogenic^+,∗^	Het	*XRCC2*	Missense	NM_005431.1	Exon 3	c.271C > T	p.R91W
NIJM6	0.25	Pathogenic	Het	*CHEK2*	Frameshift deletion	NM_007194.3	Exon 11	c.1100delC	p.T367fs
NIJM8	0.125	Likely pathogenic	Het	*CHRNA3*	Missense	NM_000743.4	Exon 5	c.877A > G	p.T293A
RUL036	0.125	Likely pathogenic^∗^	Het	*ATM*	Missense	NM_000051.3	Exon 57	c.8393C > A	p.A2798D
RUL036	0.125	Pathogenic	Het	*CTR9*	Splicing	NM_014633.4	Exon 22	c.2728-1G > A	–
RUL153	0.016	Pathogenic**^+^**	Het	*CHEK2*	Frameshift deletion	NM_007194.3	Exon 11	c.1100delC	p.T367fs
RUL39	0.0625	Likely pathogenic	Het	*IGF2R*	Missense	NM_000876.3	Exon 27	c.3718C > G	p.L1240V
RUL79	0.25	Pathogenic**^+^**	Het	*MUTYH*	Missense	NM_001128425.1	Exon 8	c.667A > G	p.I223V
F2887	0.03	Uncertain of significance^∗^	Het	*NCK1*	Missense	NM_006153.5	Exon 2	c.217G > C	p.D73H

*+, indicates the corresponding variant reported in the original publication; ^∗^, the corresponding variants that occurred in TCGA-germline database; Het, Heterozygous; Kinship of the samples applied to WES were estimated based on the pedigree structure; all the variants were shared between the two samples applied to WES per pedigree.*

In our analysis, we found that seven out of 10 pedigrees had potential co-segregated pathogenic variants in known cancer-associated genes (Table 1 and Supplementary Table S1), including *CHEK2*, *ATM*, *MRE11*, and *CTR9*, and some other cancer-associated genes, such as *IGF2R* and *CHRNA3* (Table 1 and Supplementary Table S1). Interestingly, we found that *XRCC2* (p.R91W) and *ATM* (p.A2798D) co-occurred in the ExAC TCGA-germline dataset (Table 1 and Supplementary Table S1). Furthermore, the *XRCC2* (p.R91W) was also reported in the original publication (Hilbers et al., 2012) and an independent pedigree (Park et al., 2012), which further confirmed our approach was effective. Finally, we identified a novel candidate gene, *NCK1*, from pedigree F2887 (Table 1 and Supplementary Table S1). *NCK1* (p.D73H) occurred once in about 7000 TCGA samples, but did not show up in more than 60,000 control samples (Supplementary Table S2). Generally, we succeeded in identifying potential cancer predisposition variants from eight in 10 pedigrees in the evaluation.

### Most of the Somatic and Germline Mutations in *NCK1* Were Intolerant

So far, few publications have reported the cancer predisposition role of *NCK1*. First, we inspected the *NCK1* variants in the genome aggregation database (genomAD), which contained the cancer patient cohort and provided detailed cohort information, such as non-cancer, control (Supplementary Table S2). We could therefore retrieve the allele counts and allele numbers of the corresponding variants recorded in genomAD for enrichment analysis (Supplementary Table S2). Additionally, we only focused on the high-quality variants, which were marked as a pass in both the exome and genome datasets. The *NCK1* mutations were significantly enriched in the cancer cohort, non-cancer cohort, and general cohort in comparison to the control cohort (Fisher-test; *P* < 0.001) (Supplementary Table S2).

**FIGURE 1:**
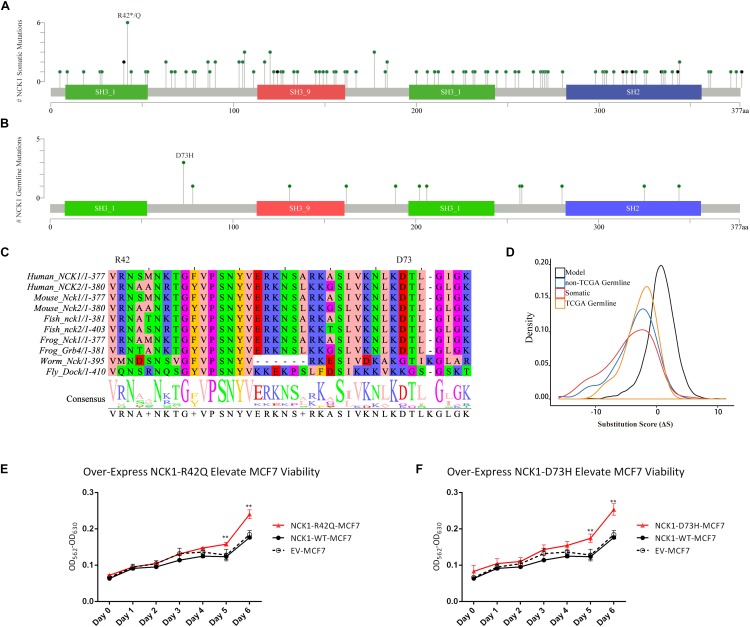
*NCK1* mutation diagram and potential functional effect. **(A)** Mutation diagram of *NCK1* collected in cBioportal (Pan-Cancer). **(B)** Mutation diagram of germline mutations in *NCK1*, including all TCGA-germline variants and *NCK1* D73H, identified in familial breast cancer pedigree (F2887). **(C)** Multiple sequence alignment of sequence flanking NCK1 D73 residue. **(D)** Distribution of substitution score (ΔS) of NCK1 based on Position Specific Score Matrix. **(E and F)** The cell viabilities in all groups of mutant over-expression assay about R42Q **(E)** and D73H **(F)** at different time points (0, 1, 2, 3, 4, 5, and 6 days). Data were expressed as mean ± standard deviation (SD) of experiments with triplicates. Asterisks indicate significant increasing of cell viability in mutant (R42Q and D73H) transfected MCF7 cells compared with wild type transfected MCF7 cells (Student’s *t*-test; *P* < 0.01). Model: random mutations generated by *in silico*, non-TCGA germline: variants collected in ExAC non-TCGA dataset; TCGA-germline: variants collected in ExAC, but not in the ExAC non-TCGA dataset; Somatic: somatic variants collected in cBioportal.

Second, we inspected the occurrence of somatic mutations in *NCK1* among pan-cancer datasets since the somatic event is another important factor involved in cancer progression. According to pan-cancer datasets, 0.3% of patients had *NCK1* somatic mutations, including 102 non-silent mutations from 97 patients, and four fusion variants impaired *NCK1* in four patients (Figure 1A). *NCK1* mutations were enriched in some cancer types, including uterine endometrioid carcinoma (*P* = 1e^−16^), stomach adenocarcinoma (*P* = 6.679e^−06^), cutaneous melanoma (*P* = 2.63e^−05^), but not BC (*P* > 0.05) (Supplementary Figure S1; Binominal test). Among these mutations, residue 42 is the most frequent in somatic, and residue 73 mutated in both the BC pedigree and the cancer cohort (Figure 1A,B). Given the rarity of the *NCK1* germline and somatic mutations, we supposed that mutations in *NCK1* might be intolerant.

To confirm that supposition, we generated a position specific score matrix (PSSM) via PSI-BLAST (Altschul et al., 1997) and predicted the damaging effect with SIFT (Ng and Henikoff, 2003) and PolyPhen2 (Adzhubei et al., 2010). *In silico*, PolyPhen2 predicted that those two were possibly damaging, and SIFT predicted that those two mutations were tolerant. Paradoxically, the residue D73 and R42 are conserved among 100 vertebrates according to MultiZ alignment (Supplementary Figure S2; Rosenbloom et al., 2015), and the residue R42 and D73 are both conserved in NCK1 and NCK2, which is the paralog of NCK1, but not conserved in the orthologs in *Caenorhabditis elegans* and *Drosophila melanogaster* (Figure 1C). According to PSSM, both germline and somatic mutations of *NCK1* were more intolerant than randomly modeling mutations (Figure 1D), and substitution score of NCK1 D73H (ΔS = −3) and NCK1 R42Q (ΔS = −1) both are negative. *In vitro*, we found that both the mutants could increase cell viability (Figure 1E,F); therefore, both the *NCK1* mutations should be deleterious.

### Role of *NCK1* Variations in Tumor Progression

Based on the “20/20” rule (Vogelstein et al., 2013), which means that more than 20 percent missense were located in recurred residues (Figure 1A,B), we supposed that *NCK1* might have an oncogenic role. According to hotspot analysis of *NCK1* somatic mutations, we found that the residue 42 turned to be a hotspot site (*P* < 0.001) (Supplementary Table S3). Indeed, NCK1-D73H and NCK1-R42Q transfected MCF7 cell lines showed significantly increased cell viability in comparison with wild type (Figure 1E,F). In addition, NCK1 contains an N-WASP activation motif (Okrut et al., 2015), where the residue D73 locates. Given this, we supposed that *NCK1* might involve in tumor invasion.

**FIGURE 2:**
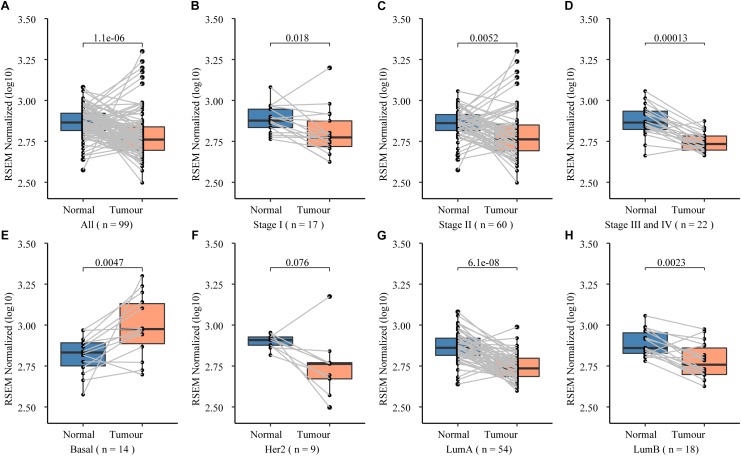
Expression spectrum of *NCK1* in 99 tumor-normal paired samples across different stage and subtypes. **(A)** All; **(B)** Stage I; **(C)** Stage II; **(D)** Stage III and IV; **(E)** basal-like; **(F)** Her2; **(G)** LumA (LuminalA); and **(H)** LumB (LuminalB).

**FIGURE 3:**
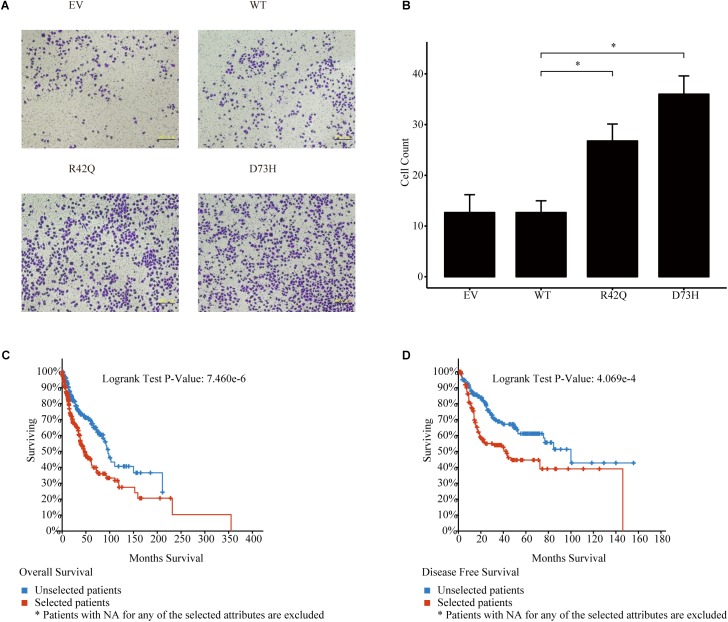
Roles of *NCK1* in tumor progression might be context dependent. **(A)** Images of MCF7 cells migrated from transwell membrane **(B)** Cell count and quantitative analysis of the migrated MCF7 cells. Patients with both *NCK1* aberrations and TP53 mutations showed a much poorer overall survival **(C)** and disease-free survival **(D)**. Selected patients: patients with both *NCK1* aberrations and *TP53* mutation. Unselected patients: patients with only *NCK1* aberrations. Scale bar: 200 μm. Data are depicted as mean ± standard deviation (SD).

To further prove that, we assessed the *NCK1* mRNA expression level among 99 tumor-normal matched samples from TCGA-BRCA. However, the expression of *NCK1* mRNA in tumor samples was significantly lower than the matched normal samples (Figure 2A), which was also observed across different tumor stages (Figure 2B–D). Mindful that BC is a molecular heterogenous cancer type, we retrieved PAM50 subtypes of the corresponding samples (Netanely et al., 2016). We found that *NCK1* was significantly upregulated in the basal-like subtype (Figure 2E). No significant difference was observed in the Her2 subtype (Figure 2F), but the expression of *NCK1* was still significantly downregulated in the Luminal A (Figure 2G) and Luminal B subtype (Figure 2H), especially in Luminal A. In this study, both NCK1-R42Q and NCK1-D73H transfected MCF cell lines, which are luminal subtypes, and showed a significantly increased invasion ability (Figure 3A,B). Recently, Morris et al. (2017) reported that the deficiency of *Nck* in MDA-MB-231, which is a basal-like subtype, could delay BC progression and metastasis, which was consistent with our results - given that *NCK1* also plays a vital role in tumor invasion. Finally, we inspected the survival status of the patients with *NCK1* variations, including CNVs, somatic mutations, and a Z-score normalized mRNA expression level, via cBioPortal (Gao et al., 2013). We found that the patients with both *NCK1* variations and *TP53* mutations had poorer overall survival (*P* < 0.05) and disease-free survival (*P* < 0.05) (Figure 3C,D). In general, the roles of *NCK1* in tumor progression could be genomic context dependent and differentiated in cancer types.

## Discussion

Intense efforts have been dedicated to identifying BC genes; however, more than 50% of familial BC heritability is still undetermined (Melchor and Benitez, 2013). Furthermore, non-BRCA1/2 familial BC patients are highly heterogeneous. For instance, we found *CHEK2* mutations from four pedigrees, including pedigree RUL153, NIJM6, NIJM8 and RUL70 (Supplementary Table S4). The *CHEK2* (p.T367fs) in pedigree NIJM8 appears to be homozygous but was only identified in one patient. Two separate *CHEK2* variants were identified from members of pedigree NIJM8 (Supplementary Table S4). In RUL70, we also identified a *CHEK2* mutation from only one patient. However, the confident predisposition variant in *XRCC2* (Table 1) identified from another *CHEK2* positive pedigree (NIJM6) further complicate the evaluation. *CHEK2* (p.T367fs) was not co-segregated across all patients in RUL153, which was explained as a phenocopy (Gracia-Aznarez et al., 2013). Although *CHEK2* (p.T367fs) is a well-known BC predisposition gene (Meijers-Heijboer et al., 2002), the co-segregation status of the variant has turned out to be negative among those pedigrees. Due to the patients in RUL70 and NIJM6, NIJM8 has been reported with a chromosome 22 gain like profile (Hilbers et al., 2013), where *CHEK2* locates, and we therefore suppose that structural variants might also contribute.

During our analysis, we also identified some likely pathogenic variants in recently established cancer predisposition genes, such as *MRE11* (Bartkova et al., 2008; Damiola et al., 2014) and *CTR9* (Hanks et al., 2014). MRE11A, encoded by *MRE11*, acting as a component of the MRN (MRE11A-RAD50-NBN) complex, which plays a vital role in DNA double-strand break repair (Yuan et al., 2012). Dysfunction of the MRN complex could promote BC invasion and metastasis (Gupta et al., 2013). In pedigree RUL036, we identified two candidate predisposition genes, including *ATM* and *CTR9*. Although the *ATM* variant occurred in the TCGA-germline dataset, multiple *in silico* tools predicted it to be benign or tolerant. *CTR9* was first reported as a Wilms tumor predisposition gene, and the mutations are almost truncated (Hanks et al., 2014). As it occurs in the Wilms tumor, we also identified a splicing site mutation in *CTR9*. Interestingly, evidence indicates that CTR9 plays an import role in regulating the estrogen signaling pathway, which promotes estrogen receptor α (ERα) positive BC progression (Zeng and Xu, 2015). In addition, we found a rare non-silent mutation in *IGF2R*. *IGF2R* is a polymorphic imprinting locus in humans (Xu et al., 1993), which indicates that individuals with *IGF2R* imprinted, might have increased cancer susceptibility (Feinberg, 1993). *CHRNA3* encodes an α type subunit of the nicotinic acetylcholine receptor. Polymorphisms in *CHRNA3* have been associated with increased smoking initiation risk and increases susceptibility to lung cancer (Hung et al., 2008). Given the heterogeneity in BC, the predisposition genes might have different disease-causative mechanisms and predisposition factors of non-BRCA1/2 pedigrees might be multifactorial, such as gene-environment interaction.

In our study, we mainly focused on gene *NCK1*, because few reports suggest the underlying predisposition role of *NCK1* mutations. As an adaptor, NCK1 mediated multiple signaling pathways, especially actin dynamic and organization involved in invadopodia formation and maturation (Stylli et al., 2009; Oser et al., 2010). The SH2 domain of NCK1 involves the recognition of cell surface receptors and transduces signals to downstream effectors (Li et al., 2001). The SH3 domain of NCK1 usually interacts with downstream effectors, most of which involves the actin cytoskeletal dynamic. For instance, NCK1 is required for EGFR-mediated cell migration and tumor metastasis (Huang et al., 2012). And the metastasis-promoting role of *NCK1* has been reported in multiple cancer types, such as colorectal cancer (Zhang et al., 2017) and BC (Morris et al., 2017). Interestingly, *NCK1* also have connections to the hotspot mutation of *PIK3CA*. Wu et al. reported that oncogenic mutations of *PIK3CA* mediate tumor cell invasion through cortactin (Wu et al., 2014), which is a partner of NCK1 in invadopodia maturation (Oser et al., 2010). Therefore, *NCK1* might be an invisible participant in tumor progression, because *NCK1* mutations rarely occur in cancer patients.

On the one hand, overexpression of *NCK1* shows oncogenic roles (Li et al., 1992), and the high expression of *NCK1*, at least in basal-like BC, contributes to tumor proliferation and metastasis (Morris et al., 2017). In our study, we identified a mutation in a motif that is involved in N-WASP activation, which is involved in invadopodia maturation (Okrut et al., 2015). Our results showed that both the NCK1 mutants (D73H and R42Q) indeed promote cell proliferation and invasion *in vitro*. We propose that *NCK1* not only contributes to cancer predisposition but is also involved in cancer progression and prognosis. In addition, our results also suggest that the tumor-promoting role of *NCK1* might be a cancer subtype dependent. On the other hand, downregulation of *NCK1* might also be pathogenic, but in different mechanisms. For instance, *Nck* degradation could prevent cancer cells from apoptosis (Li et al., 2013) and regulate actin dynamics (Buvall et al., 2013). Furthermore, *NCK1* played important roles in angiogenesis (Zhang et al., 2017; Xia et al., 2018) and even has an unexpected link to CHEK2 activation (Kremer et al., 2007).

Traditional approaches to identify underlying predisposition genes usually involves allele frequency filtering and *in silico* prediction and the sequences involved in the comparative analysis could also impact the final accuracy. Although we identified some novel candidate cancer predisposition variants, the power to confirm the predisposition role of those variants was limited. Because most of candidate cancer predisposition variants identified in our analysis turn out to be familial specific, which indicates that the power to establish a novel predisposition variant depends on an extremely large sample size (Guo et al., 2016). For instance, the variant *NCK1* (p.D73H), identified from the pedigree F2887, occurred once in about 7,000 cancer samples, but not in about 60,000 controls according to the genomAD datasets. The predisposition role of *NCK1* mutations was ignored probably because of its rare occurrence. In general, our results support *NCK1* as a candidate cancer gene; however, the underlying mechanisms requirefurther investigation. In addition, we imagine that many more cancer genes like *NCK1* might exist.

## Author Contributions

JY, LH, and XK aided in data collection and developed the concepts. JY performed the data analysis, variants evaluation, vector construction, table, created the figures, and drafted and revised the manuscript. KW aided in variants evaluation, vector construction, *in vitro* experiments, and manuscript revision. QM and HD aided in experiments design and manuscript revision. YZ and LH aided in data collection and manuscript revision. XK supervised all the study. All the authors read and approved the final manuscript.

## Conflict of Interest Statement

The authors declare that the research was conducted in the absence of any commercial or financial relationships that could be construed as a potential conflict of interest.

## Footnotes

1 http://www.bioinformatics.org/primerx/

## Supplementary Material

FIGURE S1 Summary of *NCK1* variations across TCGA studies.

FIGURE S2 Multiple sequence alignment of residue flanking R42 and D73 across 100 vertebrates.

TABLE S1 Variant filtering and classification parameters and the whole list of candidate genes.

TABLE S2 The allele count and allele number of the non-silent *NCK1* mutations retrieved from genomAD and the burden test result.

TABLE S3 Hotspot analysis of *NCK1* somatic mutations.

TABLE S4 All *CHEK2* variants identified from the pedigrees.
