# Supplementary figures and images for: Revisiting Non-BRCA1/2 Familial Whole Exome Sequencing Datasets Implicates NCK1 as a Cancer Gene
Source: Front Genet. 2019 Jun 4;10:527. doi: 10.3389/fgene.2019.00527 (PMC6557995; doi:10.3389/fgene.2019.00527)

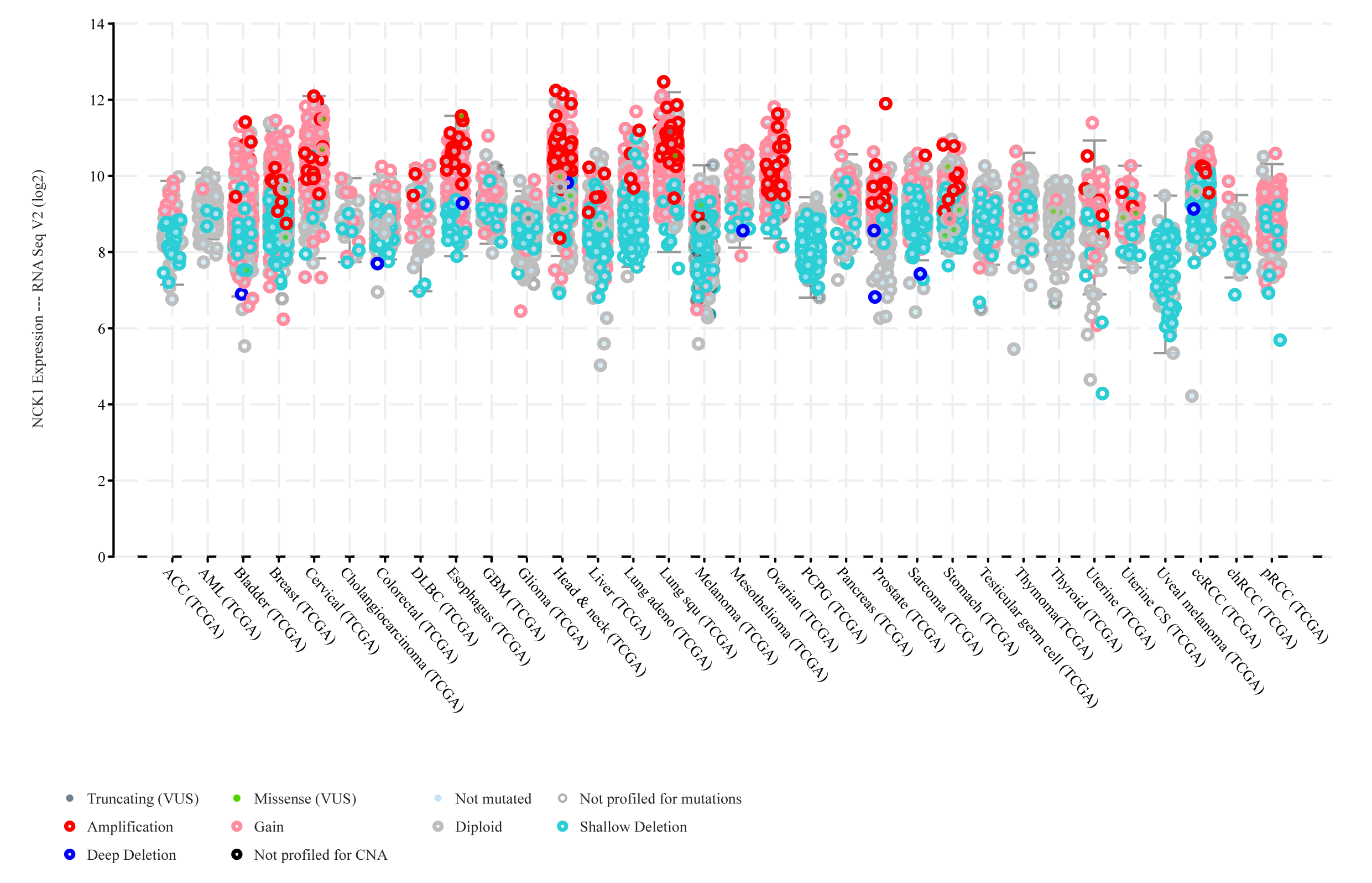

Supplement: FIGURE S1 — Summary of NCK1 variations across TCGA studies. [file Image_1.TIF]

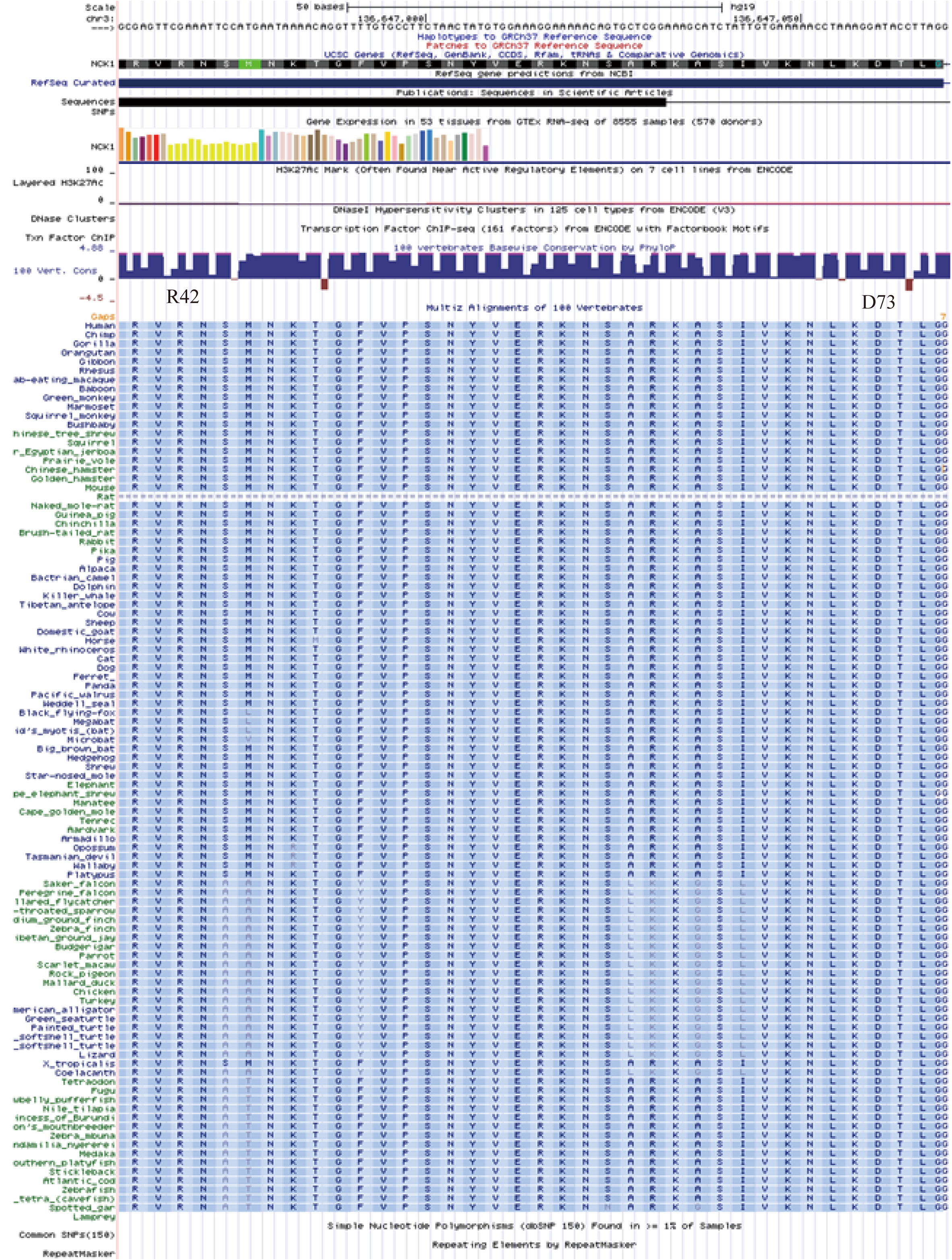

Supplement: FIGURE S2 — Multiple sequence alignment of residue flanking R42 and D73 across 100 vertebrates. [file Image_2.TIF]
